# Network pharmacology based virtual screening of active constituents of *Prunella vulgaris* L. and the molecular mechanism against breast cancer

**DOI:** 10.1038/s41598-020-72797-8

**Published:** 2020-09-25

**Authors:** Xiaobo Zhang, Tao Shen, Xin Zhou, Xuehua Tang, Rui Gao, Lu Xu, Long Wang, Zubin Zhou, Jingjing Lin, Yuanzhang Hu

**Affiliations:** 1 School of Basic Medicine, Chengdu University of Traditional Chinese Medicine, Chengdu, 611137 China; 2 Academic Department, Zhuhai Ebang Pharmaceutical Co., Ltd, Zhuhai, 519040 China; 3 College of Information Engineering, Chengdu University of Traditional Chinese Medicine, Chengdu, 611137 China

**Keywords:** Cancer, Computational biology and bioinformatics

## Abstract

*Prunella vulgaris* L, a perennial herb widely used in Asia in the treatment of various diseases including cancer. In vitro studies have demonstrated the therapeutic effect of *Prunella vulgaris* L. against breast cancer through multiple pathways. However, the nature of the biological mechanisms remains unclear. In this study, a Network pharmacology based approach was used to explore active constituents and potential molecular mechanisms of *Prunella vulgaris* L. for the treatment of breast cancer. The methods adopted included active constituents prescreening, target prediction, GO and KEGG pathway enrichment analysis. Molecular docking experiments were used to further validate network pharmacology results. The predicted results showed that there were 19 active ingredients in *Prunella vulgaris* L. and 31 potential gene targets including AKT1, EGFR, MYC, and VEGFA. Further, analysis of the potential biological mechanisms of *Prunella vulgaris* L. against breast cancer was performed by investigating the relationship between the active constituents, target genes and pathways. Network analysis showed that *Prunella vulgaris* L. exerted a promising preventive effect on breast cancer by acting on tumor-associated signaling pathways. This provides a basis to understand the mechanism of the anti-breast cancer activity of *Prunella vulgaris* L.

## Introduction

*Prunella vulgaris* L. is a perennial herbaceous plant in the genus prunella^1^. It is a traditional Chinese medicine widely used for the treatment of inflammation, eye pain, headache, and cancer^2,3^. Modern pharmacological studies suggest that *Prunella vulgaris* L. possesses antiviral, antibacterial, anti-inflammatory, immunoregulatory, anti-oxidative and anti-tumor functions^4,5^. Lee et al.^6^ reported that ursolic acid in *Prunella vulgaris* L. provides the anticancer effects. This study also showed marginal cytotoxicity in KB cells, human colon cancer cells (HCT-8) and breast tumor cells (MCF-7). Other related studies have reported that oral administration of *Prunella vulgaris* L. with taxane prevents breast cancer progression as well as reduces its side effects^7^. In addition, crude extracts of *Prunella vulgaris* L. are reported to inhibit the proliferation of breast cancer cells and also induce their apoptosis^8^. Triterpenoids in *Prunella vulgaris* L. have shown selective inhibitory effects on breast cancer cells and normal breast cells. These approaches, however, fail to address the anti-breast cancer mechanism of *Prunella vulgaris* L, while the active ingredients and targets also remain unclear. In vitro, experimental studies have validated the anti-cancer activity of most of the active ingredients in *Prunella vulgaris* L. However, the underlying molecular mechanisms are poorly understood.

Network pharmacology is a new drug discovery approach created by Hopkins in 2007 and integrates systematic medicine with information science^9^. It emphasizes on the concept of “network target, multicomponent therapeutics”^10,11^, shifting the paradigm from the concept of one gene, one target, and one disease. Network pharmacology is a powerful method used to study the synergistic actions and underlying mechanisms of traditional medicine^12–14^.

DL is a qualitative character used to describe the physical and chemical properties of drugs, for example, solubility, stability, and bioavailability are some of the important properties^15^. Additionally, it is regarded as a property to use when evaluating the clinical efficacy of a compound since it has a guiding role in the prediction of new drugs^16^.

ADME is an abbreviation in pharmacokinetics representing absorption, distribution, metabolism, and excretion and it is essential in drug discovery^17^. Caco-2 cell model (Caco-2), a human clone of colorectal adenocarcinoma cell, is used to predict the constituent’s intestinal absorption. Human Intestinal Absorption (HIA) is important in the identification of potential drug candidates and it refers to the sum of bioavailability and absorption assessed from the ratio of excretion or cumulative excretion in urine, bile, and feces. It is used to evaluate the absorption capacity of drugs in humans. Plasma Protein Binding (PPB) affects the function, distribution, and efficacy of drugs, and PPB rate is used to predict the distribution of drugs in humans.

Molecular docking has shown potential applications in the field of computer-aided drug research, especially in the development of new treatment targets against diseases caused by genetic mutations^18,19^. Moreover, the method has emerged as a powerful tool in the study of drug active sites hence playing a significant role in natural product research.

**Figure 1:**
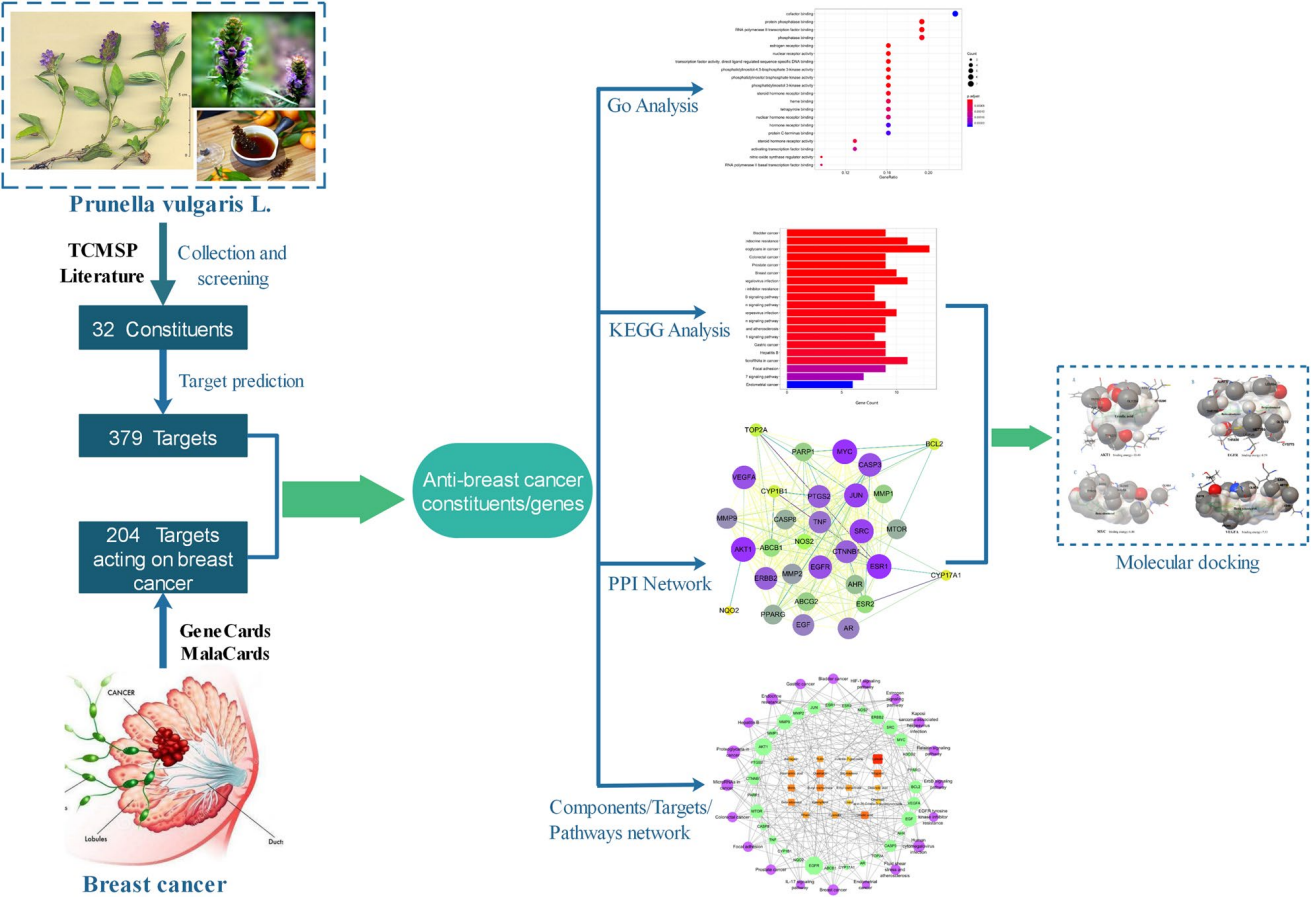
Flow chart of the network pharmacology based study. *Go* Gene Ontology, *KEGG* Kyoto encyclopedia of genes and genomes, *PPI* protein–protein interaction.

In this study, the active constituents of *Prunella vulgaris* L. and the potential mechanism underlying its anti-breast cancer effect were explored using network pharmacology. Several databases were used to predict *Prunella vulgaris* L. target sites and GO biological process analysis and KEGG pathway enrichment analysis were used to investigate the possible mechanisms involved in the anti-breast cancer effect of *Prunella vulgaris* L. Molecular docking of key targets was used to validate network pharmacology of selected active constituents. The flow chart of the study is shown in Fig. 1.

## Results

### Filtering of active constituents of *Prunella vulgaris* L

A total of 60 constituents of *Prunella vulgaris* L. were retrieved from the TCMSP database while 18 constituents were retrieved from literature (Supplementary Table 1). Constituents with DL ≥ 0.18 were retained as active ingredients and 41 chemical components matched the threshold (Supplementary Table 2). The potential targets of the identified components were submitted to the preADMET website where further screening was performed based on ADME parameters, Caco-2, HIA and PPB. The screening results showed that 31 constituents were considered to be biologically active in vivo with the major active compounds being flavonoids, triterpenes, and phenolic acids. These findings were consistent with previously reported anti-cancer active components in *Prunella vulgaris* L^1,20–22^. Although the HIA and PPB of rutin predicted by preADMET were lower, it has been reported with significant biological activity^23^, this constituent was temporarily included for further network pharmacology experiments. Table 1 presents the DL and partial ADME values of the 32 final filtration constituents.

**Table 1:** DL and partial ADME values of the 32 DL filtered components.

Components	DL	Caco-2	HIA	PPB
Oleanolic acid-28-O-beta-D-glucopyranoside	0.54	20.67	83.27	97.72
Cyanidol	0.92	0.66	66.71	100.00
Oleanolic acid	0.37	21.89	96.00	100.00
Sitogluside	0.51	25.23	90.03	100.00
Beta-sitosterol	0.88	52.37	100.00	100.00
Rutin	1.10	7.91	2.86	43.90
Arjunglucoside I	0.62	20.13	30.74	76.25
Kaempferol	0.77	9.58	79.44	89.61
Stigmasterol	0.73	52.34	100.00	100.00
Ursolic acid	0.65	21.86	96.00	100.00
Δ7-stigmasterol	0.40	52.27	100.00	100.00
Astragalin	0.80	11.15	25.17	57.58
Luteolin	0.86	4.54	79.43	99.72
Vulgarsaponin B	0.32	20.24	76.98	93.7
Nigaichigoside F1	0.73	19.96	30.78	71.85
Poriferasterol monoglucoside	0.32	25.16	90.57	100.00
Poriferasterol monoglucoside_qt	0.57	54.6	100.00	100.00
Sericoside	0.62	20.13	30.74	76.25
Stigmast-7-enol	0.30	52.37	100.00	100.00
Morin	0.87	17.10	63.49	91.63
Luteolin-7-glucoside	0.86	52.37	100.00	100.00
Quercetin	0.93	3.41	63.49	93.24
Rosmarinic acid	0.63	20.72	62.49	86.24
2α,3α-dihydroxyursa-12-en-28-oic acid	0.56	21.26	94.28	99.22
Stigmasterol-3-O-β-d-glucoside	0.32	25.16	90.57	100.00
Uvaol	0.18	24.66	94.41	100.00
Lupenone	0.37	49.54	100.00	100.00
Wogonin	0.25	4.28	93.04	90.45
Acacetin-7-O-β-d-glucopyranoside	0.72	7.73	65.90	68.78
Ethyl rosmarinate	0.62	20.52	78.87	86.96
Butyl rosmarinate	0.67	20.54	82.15	90.42
Rhein	0.79	2.84	82.96	88.52

### Screening of anti-breast cancer targets

A total of 379 potential targets were obtained from 32 active constituents retrieved from STITCH and Swiss Target Prediction databases (Supplementary Table 3). Further, a total of 204 gene targets associated with breast cancer were retrieved from Malacards and GeneCards (supplementary Table 4). Common targets of both breast cancer and the chemical constituents were considered potential targets. 31 potential anti-breast cancer genes of *Prunella vulgaris* L. are shown in Table 2.

**Table 2:** 31 potential anti-breast cancer target genes of active components.

UniProt ID	Protein name	Gene name
P04626	Receptor tyrosine-protein kinase erbB-2	ERBB2
Q9SAD4	Ethylene-responsive transcription factor ESR1	ESR1
P31749	RAC-alpha serine/threonine-protein kinase	AKT1
P15692	Vascular endothelial growth factor A	VEGFA
P35222	Catenin beta-1	CTNNB1
P01106	Myc proto-oncogene protein	MYC
P10275	Androgen receptor	AR
P01375	Tumor necrosis factor	TNF
P00533	Epidermal growth factor receptor	EGFR
Q14790	Caspase-8	CASP8
Q92731	Estrogen receptor beta	ESR2
P42345	Serine/threonine-protein kinase mTOR	MTOR
P10415	Apoptosis regulator Bcl-2	BCL2
P12931	Proto-oncogene tyrosine-protein kinase Src	SRC
P05093	Steroid 17-alpha-hydroxylase/17,20 lyase	CYP17A1
P11388	DNA topoisomerase 2-alpha	TOP2A
P01133	Pro-epidermal growth factor	EGF
P03956	Interstitial collagenase	MMP1
P16083	Ribosyldihydronicotinamide dehydrogenase [quinone]	NQO2
P42574	Caspase-3	CASP3
P35354	Prostaglandin G/H synthase 2	PTGS2
P14780	Matrix metalloproteinase-9	MMP9
P08253	72 kDa type IV collagenase	MMP2
Q16678	Cytochrome P450 1B1	CYP1B1
Q9UNQ0	ATP-binding cassette sub-family G member 2	ABCG2
P08183	Multidrug resistance protein 1	ABCB1
P05412	Transcription factor AP-1	JUN
P35228	Nitric oxide synthase	NOS2
P37231	Peroxisome proliferator-activated receptor gamma	PPARG
P35869	Aryl hydrocarbon receptor	AHR
P09874	Poly [ADP-ribose] polymerase 1	PARP1

### GO and KEGG analysis

**Figure 2:**
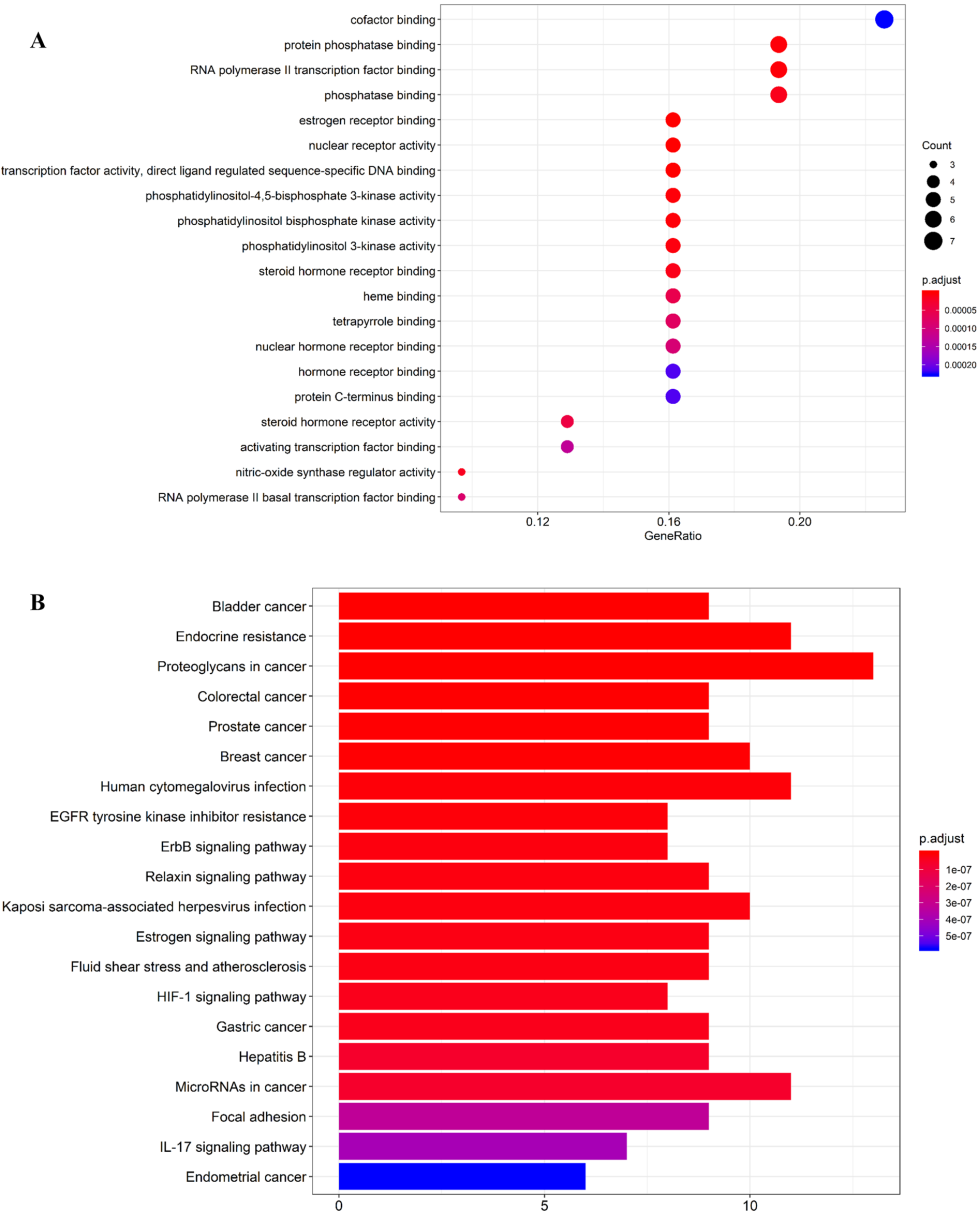
Top 20 GO enrichments and KEGG pathways annotation. (**A**) GO enrichment. X-axis is enrichment gene ratio, Y-axis is molecular function or biological process. Bubble size represents the number of genes involved in the GO enrichment. Color represents the adjusted p-value, the darker the color, the smaller the adjusted p-value. (**B**) KEGG pathway enrichment. X-axis is enrichment gene count, Y-axis is KEGG pathway, and the color of bar chart represents the adjusted p-value.

Bioconductor package in R software was used to construct the top 20 main pathways by GO analysis (Fig. 2A) and KEGG analysis (Fig. 2B). GO functional analysis predicted that the key targets of *Prunella vulgaris* L. are mainly involved (supplementary table 5) in estrogen receptor binding, steroid hormone receptor binding, steroid hormone receptor activity, and so forth. KEGG pathway analysis was used to determine relevant signaling pathways associated with the anti-breast cancer effect of *Prunella vulgaris* L. The following processes had the highest number of genes: Proteoglycans in cancer (13), Endocrine resistance (11), Human cytomegalovirus infection (11), MicroRNAs in cancer (11), Breast cancer (10), and Kaposi sarcoma-associated herpesvirus infection (10). From the 20 KEGG signaling pathways results (supplementary table 6), the significantly enriched genes were EGF, AKT1, EGFR, ERBB2, SRC, MTOR, MYC, BCL2, JUN, VEGFA, MMP9, and CTNNB1.

### Compounds target network construction

**Figure 3:**
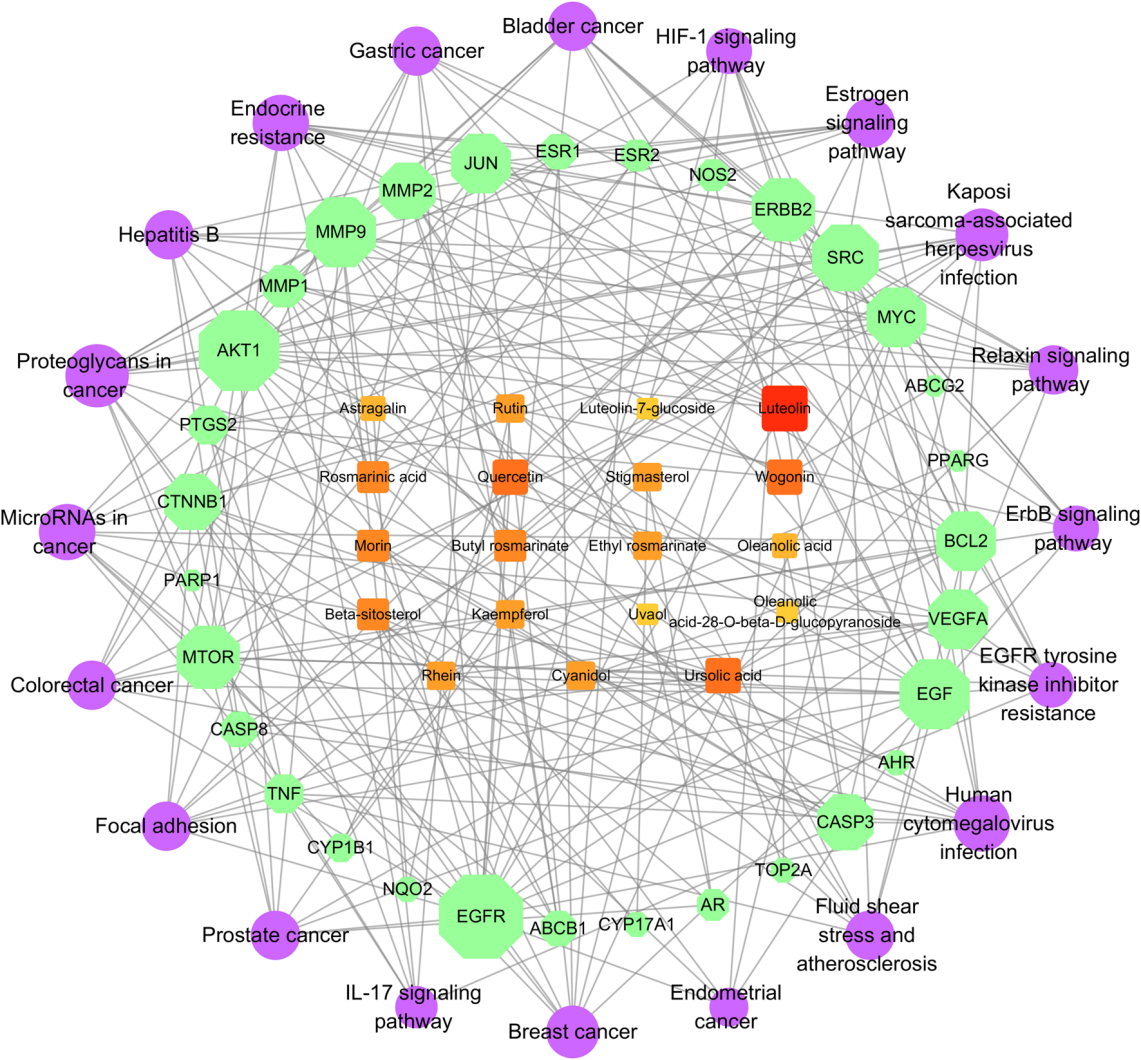
Network diagram of active components/target genes/enrichment pathways. The orange square nodes represent the active components, the green octagon nodes represent the target genes while the purple circle nodes represent the pathways. Nodes size are proportional to their degree.

A total of 32 satisfactory chemical constituents were gained from *Prunella vulgaris* L. Among the 32 compounds, 13 compounds could not be successfully predict anti-breast cancer genes, hence only 19 constituents were retained. Further, 19 constituents, 31 potential anti-breast cancer genes and the top 20 pathways (P ≤ 0.01) with the highest number of genes were selected to construct the compound-target-pathway network diagram (Fig. 3). Each compound corresponded to multiple targets in the network diagram. This reveales that multiple targets may result in a synergistic effect when *Prunella vulgaris* L. plays a role in anti-breast cancer. The degree of the 19 active components in the compound-target-pathway network was analyzed (Table 3). In the table, flavonoids and triterpenes have a relatively higher degree, while anthraquinones and saponins are relatively lower. The following 8 components were retained for further docking experiments: three higher degree flavonoids, namely luteolin, quercetin‚ and wogonin; one triterpene component, namely ursolic acid; one phenolic acid component, namely rosmarinicacid; one sterol component, namely beta-sitosterol; one anthraquinone component, namely rhein; and one saponin component, namely astragalin.

**Table 3:** Degree of 19 active components analyzed by function tool in Cytoscape.

Sorts	Chemical name	Degree
Flavonoid	Luteolin	8
Flavonoid	Quercetin	5
Flavonoid	Wogonin	5
Flavonoid	Morin	4
Flavonoid	Cyanidol	3
Flavonoid	Rutin	3
Flavonoid	Kaempferol	3
Flavonoid	Luteolin-7-glucoside	1
Triterpene	Ursolic acid	5
Triterpene	Oleanolic acid	2
Triterpene	Uvaol	1
Triterpene	Oleanolic acid-28-O-beta-D-glucopyranoside	1
Phenolic acid	Rosmarinic acid	4
Phenolic acid	Butyl rosmarinate	4
Phenolic acid	Ethyl rosmarinate	3
Sterol	Beta-sitosterol	4
Sterol	Stigmasterol	3
Anthraquinone	Rhein	3
Saponin	Astragalin	2

### PPI network construction and molecular docking analysis

**Figure 4:**
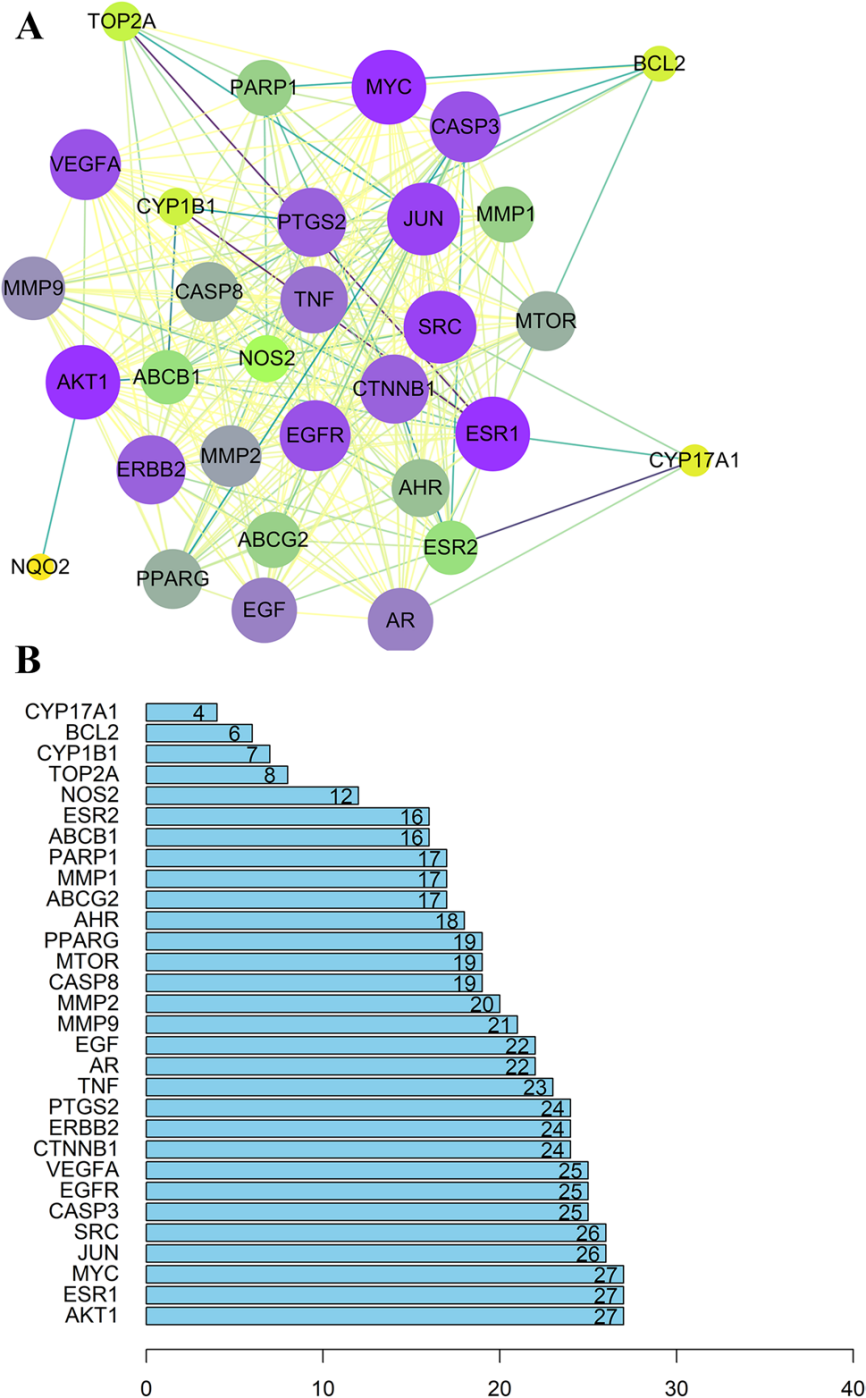
(**A**) The protein–protein interaction (PPI) network. (**B**) The bar plot of the protein–protein interaction (PPI) network. The nodes represent the targets, the size shows their degree in the network.

31 target genes associated with anti-breast cancer activity were imported into the STRING database for PPI network construction (Supplementary table 7). The nodes in the PPI network represent the interrelationships during the development of breast cancer (Fig. 4A). Analyze tool in Cytoscape was applied to analyze the PPI diagram^24^, AKT1 (27), ESR1 (27), MYC (27), JUN (26), SRC (26), CASP3 (25), EGFR (25), and VEGFA (25) showed a higher degree (Fig. 4B). Comparing the results with those provided by KEGG analysis, four target genes, AKT1, EGFR, MYC, and VEGFA were selected for molecular docking experiments.

**Figure 5:**
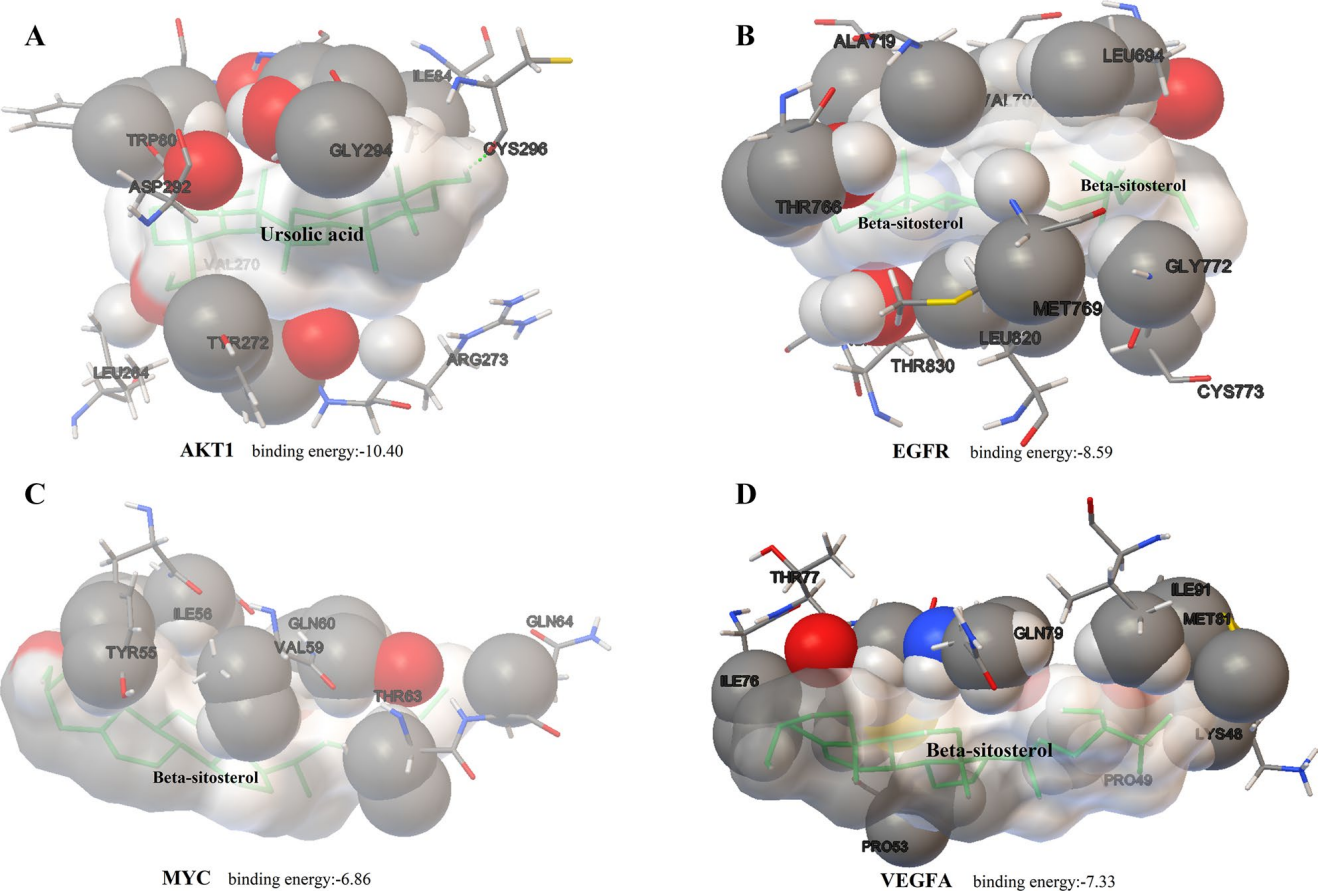
The docking complex of four targets and their strongest binding components. The green sticks represent the ligand while the spheres represent the protein structure, the active site residues are shown. (**A**) AKT1. (**B**) EGFR. (**C**) MYC. (**D**) VEGFA.

From the Protein Data Bank (PDB) database, 3O96^25^, 4HJO^26^, 5I4Z^27^, and 4KZN^28^ were identified as the protein structures of the four key targets highlighted above for molecular docking experiments. Resveratrol^29^, erlotinib^26^, acetylsalicylic acid^30^, and minocycline^31^ were selected as positive control drugs of AKT1, EGFR, MYC, and VEGFA, respectively. Previous studies have reported the four target proteins, hence the Drugbank database was used to retrieve information on their active binding sites to inhibitors. The Grid Box parameters in AutoDockTools were set as following: 3O96, grid center 6.0 − 7.0 15.0, number of points in xyz (NPTS) 50 50 50, spacing 0.375; 4HJO, grid center 40 40 40, NPTS 24 9 1, spacing 0.375; 5I4Z, grid center 35 33 9, NPTS 80 80 80, spacing 0.375; 4KZN, grid center 7 − 5 5, NPTS 126 126 126, spacing 0.375. The docking conditions were similar after 10 times docking and binding energy was used as an important criterion for constituents screening (Table 4). Clusters with the highest conformation and maximum absolute value of binding energy were selected. Figure 5 presents the docking complex of the four targets together with their strongest binding components.

**Table 4:** Binding energy of eight active components and positive control drugs.

Compound	Binding Energy/(kcal mol^−1^ )			
	AKT1	EGFR	MYC	VEGFA
Luteolin	− 7.05	− 7.84	− 5.96	− 6.28
Quercetin	− 7.77	− 7.81	− 5.74	− 5.70
wogonin	− 7.50	− 7.14	− 5.89	− 5.90
Ursolic acid	− 10.40	− 7.56	− 6.65	− 7.11
Rosmarinic acid	− 7.15	− 7.22	− 4.50	− 4.93
Beta-sitosterol	− 10.17	− 8.59	− 6.86	− 7.33
Rhein	− 7.61	− 7.62	− 5.99	− 5.85
Astragalin	− 8.18	− 7.47	− 5.27	− 4.87
Resveratrol	− 6.61	–	–	–
Erlotinib	–	− 6.93	–	–
Acetylsalicylic acid	–	–	− 4.57	–
Minocycline	–	–	–	− 6.07

## Discussion

In recent years, natural product research has gained increased attention^32,33^. The network pharmacology-based approach promotes an understanding of the complex interactions between drugs and their targets and the potential mechanisms of action^34–36^. Similarly, the complexity of drug development from natural sources introduces methodological problems^37^. The development of new drug candidates is often limited by the lack of absorption, distribution, metabolism, and excretion (ADME) propertiese, and these high-cost nature presents a more difficult drug development process^38^. Therefore, ADME based screening has gained considerable attention from pharmaceutical scientists in drug discovery^39^.

*Prunella vulgaris* L. is a medicinal herb that is reported to possess antibacterial, anti-inflammatory and immunoregulatory effects^40^. In clinical practice, *Prunella vulgaris* L. is mainly prescribed for acute conjunctivitis, thyroid dysfunction, breast hyperplasia, and breast cancer. In the current study, screening results indicated that flavonoids and triterpenes in *Prunella vulgaris* L. were the most active constituents followed by phenolic acids. Previous studies on the anticancer activity of *Prunella vulgaris* L. focuses on the role of triterpenes and phenolic acids while limited research focuses on flavonoids, sterols or anthraquinones^20,41,42^. Studies on flavonoids such as luteolin, wogonin, morin, and kaempferol, report that they suppress the growth and invasion of human breast cancer cells^43–46^. Beta-sitosterol, a sterol compound which activates the Fas signaling is reported to induce apoptosis in human breast adenocarcinoma cells (MCF-7 and MDA-MB-231)^47^. Rhein in anthraquinones induces apoptosis in breast cancer cells (MCF-7 and SK-Br-3) through the NF-kappaB/P53 signalling pathway^48^. These flavonoids components were successfully filtered using the method of DL and ADME property screening.

As shown in GO analysis result, Estrogen receptor binding was ranked as No. 1, which indicated that this receptor might be one of the main drug targets for the treatment of breast cancer. It’s reported that estrogen receptor (ER) expresses in approximately 75% of breast cancers, modulating ER action has improved the survival of patients with ER-positive breast cancer^49^. Usually, the binding of estrogen to nuclear ERs, which then form dimers and bind to estrogen response elements in the regulatory regions of estrogen-responsive genes to alter gene expression.

KEGG analysis revealed that multiple gene targets of *Prunella vulgaris* L. served important roles in several cancer-related pathways, including the Breast cancer, ErbB signaling pathway, and Estrogen signaling pathway. As is well known, breast cancer pathogenesis is extremely complicated. Several environmental factors are known to increase the risk of breast cancer by interacting with human genes, immunity, and hormone secretion. Beatson in 1986 was the first to report a link between estrogen and breast cancer^50^. In this study, women with metastatic breast cancer before menopause showed tumor regression after bilateral oophorectomy. Subsequent studies further demonstrated that estrogen binds to ERs which directly interacts with membrane receptors (such as IGFR, EGFR, and HER2) and key signaling molecules (Shc) to activate the major second messenger and MAPK, PI3K/AKT pathways and to promote the proliferation, growth, and survival of tumor cells. Therefore, inhibition of the estrogen signaling pathway (blocking estrogen production or inhibiting ER function) is one of the effective methods in breast cancer treatment.

ErbB-2 is a member of the ErbB family and plays a vital role in breast cancer development. Previous studies demonstrate that approximately 15–20% of all diagnosed breast cancers show overexpression of ErbB-2 (MErbB-2) on the cell membrane^51^. Upon binding to the ligand, ErbB forms homodimers (ErbB-2) or heterodimers (ErbB-3), activates downstream signaling cascades and transduces the ErbBs effects. Two key signal transduction pathways that are activated include MAPK and PI3K/AKT. Upon activation, the MAPK pathway results in gene transcription that leads to cellular proliferation, migration, and angiogenesis. The PI3K/AKT pathway causes downstream cell survival and inhibition of apoptosis. Crosstalk between these signaling pathways accelerates the growth and metastasis of breast cancer. Pathway analysis also indicated that *Prunella vulgaris* L. exerts anti-breast cancer effects by inhibiting key targets in the estrogen pathway and ErbB pathways, such as AKT1, EGFR, and MYC.

Among the four targets chosen for molecular docking experiments, AKT1, EGFR, and MYC were reported as key proteins in both the ErbB signaling pathway and the estrogen signaling pathway. Ursolic acid and Beta-sitosterol were successful docked to those four target proteins with a higer binding energy compared with other components. It showed that Ursolic acid could bind to AKT1/VEGFA, then inhibited breast cancer growth through ErbB or Estrogen pathway. Beta-sitosterol bound to EGFR/MYC, inhibiting breast cancer growth through ErbB or Estrogen pathway. Molecular docking results also revealed that eight of the higher active compounds had stronger binding energies than the positive control drugs (Resveratrol, Erlotinib, Acetylsalicylic acid). These findings validate the reliability of the active ingredients screened by network pharmacology and their interaction with breast cancer targets.

Flavonoids, sterols, and anthraquinones are reported to play a crucial role in the anti-breast cancer process. Network analysis further reveals that *Prunella vulgaris* L. produces therapeutic effects on breast cancer by inhibiting key targets in the ErbB signaling pathway and estrogen signaling pathways, such as AKT1, EGFR, and MYC. Although we have described some interesting data, there is a need for further experiments to validate these findings.

## Methods

### Screening for active constituents

Information on all the constituents was obtained from literature and traditional Chinese Medicine systems pharmacology (https://tcmspw.com/tcmsp.php). “Prunellae Spica” was used as a keyword in TCMSP search while a literature search was carried out on Pubmed, CNKI database, and Google Scholar. The chemical structures were retrieved from PubChem (https://pubchem.ncbi.nlm.nih.gov/) and ChemSpider (https://www.chemspider.com/). For structures not present in the database, TCMSP was refereed to and original research articles.

DL is provided by the molsoft website (https://www.molsoft.com/mprop/) for use in predicting potential constituents. DL ≥ 0.18 was assigned as the criteria for screening act active constituents. Selected compounds (DL ≥ 0.18) were imported into preADMET (https://preadmet.bmdrc.kr/) and screened by Caco-2, HIA and PPB. The active ingredients which don’t meet this requirement but have an obvious biological activity were still considered.

### Target genes screening

STITCH (https://stitch.embl.de/) and Swiss Target Prediction (https://www.swisstargetprediction.ch/) databases were used to retrieve the gene targets for active ingredients. This was achieved by uploading the screened components to the STITCH database, selecting the Homo sapiens for the species, and collecting the targets with a combined-score ≥ 0.7. The smiles number of each component was entered into the Swiss Target Prediction online platform. Target's prediction was performed by structural similarity using a reverse pharmacophore matching method, and a target with probability ≥ 0.7 was selected.

### Potential target genes for breast cancer

The target genes retrieved from the two databases were merged. Standardization of the gene name and definition of the species as "human" was performed using the UniProtKB function in the UniProt (https://www.uniprot.org/) database. GeneCards (https://www.genecards.org/) and MalaCards (https://www.malacards.org/), the human gene database, were used to retrieve breast cancer-related genes. The keywords used in the search were limited to "breast cancer" and "mammary carcinoma". The targets obtained were compared to those retrieved earlier and target genes linked to breast cancer were selected.

### Pathway and functional enrichment analysis

KEGG analysis is one of the most prominent in network pharmacology, which interprets pathways target genes. It is beneficial to understand the mechanisms of drug action in diseases^52^. We carried out GO enrichment analysis and KEGG pathway annotation for predicting targets of *Prunella vulgaris* L. on breast cancer by Bioconductor, a data package in R software (version 3.6.1)^53^. Adjusted p-value ≤ 0.01 was chosen, and the top 20 GO enrichments or KEGG pathways with higher counts were analyzed.

### Network construction

Network analysis was performed to understand the mechanism of *Prunella vulgaris* L. in breast cancer. The network was established and visualized by Cytoscape 3.7.1 software. Active compounds and target genes were represented by nodes in the network. The edges were used to indicate an interaction between the compounds and the targets. Analyze tool in Cytoscape was employed to calculate Degree, a topological parameters which shows the importance of component/target/pathway in the network.

### PPI Network construction and molecular docking experiment

The PPI network of *Prunella vulgaris* L. on breast cancer was constructed using the STRING database in combination with the Network Analyzer plugin of Cytoscape, combined-score in STRING was set to 0.4 or greater. The key targets were prepared for molecular docking.

The crystal structures of candidate targets were downloaded from RCSB Protein Data Bank (https://www.pdb.org/) and imported into AutoDockTools 1.5.6 for docking. PyMol (version 1.7.2.1) was used to process the protein, including removing the ligands, correcting protein structure, and removing water. Similar docking conditions were used and the Lamarckian genetic algorithm chosen to calculate the binding energy.

## Supplementary information

Supplementary file1

## Data Availability

The datasets supposing the current study are available in public database from TCMSP, STITCH, Swiss Target Prediction, GeneCards, MalaCards, STRING, DrugBank, and PDB.
