# Supplementary material for: Network pharmacology based virtual screening of active constituents of Prunella vulgaris L. and the molecular mechanism against breast cancer
Source: Sci Rep. 2020 Sep 25;10:15730. doi: 10.1038/s41598-020-72797-8 (PMC7519149; doi:10.1038/s41598-020-72797-8)
Supplement: Supplementary file 1 — Supplementary file1 [file 41598_2020_72797_MOESM1_ESM.pdf]

Network pharmacology based virtual screening of active constituents of *Prunella vulgaris* L. and the molecular mechanism against breast cancer

Author list:

Xiaobo Zhang[1], Tao Shen[1*], Xin Zhou[1], Xuehua Tang[2], Rui Gao[1], Lu Xu[1], Long Wang[1], Zubin Zhou[1], Jingjing Lin[1], Yuanzhang Hu[3]

1 School of Basic Medicine, Chengdu University of Traditional Chinese Medicine, Chengdu, 611137 China
2 Academic Department, Zhuhai Ebang Pharmaceutical Co., Ltd. Zhuhai, 519040 China
3 College of Information Engineering, Chengdu University of Traditional Chinese Medicine, Chengdu, 611137 China

* Corresponding author
Tao Shen, School of Basic Medicine, Chengdu University of Traditional Chinese Medicine, Chengdu, 611137 China. Email: shentaotcm@aliyun.com

Supplementary table 1 constituents from TCMSP and Literature

**Components**	**DL**(by molsoft)	**Scourse**
1,8-cineole	-1.06	TCMSP
(-)-nopinene	-1.41	TCMSP
CAM	-0.62	TCMSP
EIC	-0.41	TCMSP
myristic acid	-0.33	TCMSP
Oleanolic acid-28-O-beta-D-glucopyranoside	0.54	TCMSP
Myrcene	-1.30	TCMSP
cyanidol	0.92	TCMSP
endo-fenchol	-1.36	TCMSP
caffeic acid	-0.02	TCMSP
Skimmetin	-0.44	TCMSP
oleanolic acid	0.37	TCMSP
l-alpha-Fenchone	-1.03	TCMSP
Ethyl caffeate	-0.12	TCMSP
lauric acid	-0.33	TCMSP
Sitogluside	0.51	TCMSP
beta-sitosterol	0.88	TCMSP
Amyrin	-0.23	TCMSP
esculetin	-1.13	TCMSP
Docosanoate	-0.77	TCMSP
Scopoletol	-1.18	TCMSP
rutin	1.10	TCMSP
kaempferol	0.77	TCMSP
Spinasterol	0.06	TCMSP
Hyperin	0.89	TCMSP
Stigmasterol	0.73	TCMSP
(2R,3R,4S,5S,6R)-2-[[(3S,5S,9R,10S,13R,14R,17R)-17-[(E,2R,5S)-5-ethyl-6-methylhept-3-en-2-yl]-10,13-dimethyl-2,3,4,5,6,9,11,12,14,15,16,17-dodecahydro-1H-cyclopenta[a]phenanthren-3-yl]oxy]-6-(hydroxymethyl)oxane-3,4,5-triol	0.03	TCMSP
delphinidin	-1.16	TCMSP
malvidin	-0.78	TCMSP
ursolic acid	0.65	TCMSP
Melissic acid	-0.33	TCMSP
[(3S)-3,7-dimethylocta-1,6-dien-3-yl] acetate	-1.06	TCMSP
Δ7-stigmasterol	0.40	TCMSP
Astragalin	0.80	TCMSP
luteolin	0.86	TCMSP
Montanic acid	-0.33	TCMSP
lignoceric acid	-0.33	TCMSP
oleic acid	-0.08	TCMSP
Malvidin-3,5-diglucoside	0.50	TCMSP
Peonin	0.35	TCMSP
peonidin	-0.31	TCMSP
Vulgarsaponin B	0.32	TCMSP
Vulgaxanthin-I	0.64	TCMSP
arjunglucoside I	0.62	TCMSP
isoquercetrin	0.89	TCMSP
niga-chigoside F1	0.73	TCMSP
poriferasterol monoglucoside	0.32	TCMSP
poriferasterol monoglucoside_qt	0.57	TCMSP
sericoside	0.62	TCMSP

stigmast-7-enol	0.30	TCMSP
palmitic acid	-0.33	TCMSP
(R)-(-)-alpha-Phellandrene	-1.24	TCMSP
morin	0.87	TCMSP
p-coumaric acid	-0.74	TCMSP
isoorientin	0.84	TCMSP
stearic acid	-0.33	TCMSP
HEXATRIACONTANE	-1.04	TCMSP
luteolin-7-o-glucoside	0.86	TCMSP
Ethylpalmitate	-1.03	TCMSP
quercetin	0.93	TCMSP
2α,3α-dihydroxyursa-12-en-28-oic acid	0.56	Literature[1]
Acacetin-7-O-β-d-glucopyranoside	0.72	Literature[2]
Butyl rosmarinate	0.67	Literature[3]
Ethyl rosmarinate	0.62	Literature[3]
Kaempferol-3-O-rutinoside	1.02	Literature[1]
Lupenone	0.37	Literature[4]
p-Coumaric acid	-0.74	Literature[3]
Protocatechualdehyde	-0.66	Literature[5]
Quercetin-3-O-β-d-galactoside	0.89	Literature[6]
Rhein	0.79	Literature[7]
Rosmarinic acid	0.63	Literature[3]
Salviaflaside	0.39	Literature[8]
Stigmasterol-3-O-β-d-glucoside	0.32	Literature[9]
Tanshinone I	-1.02	Literature[7]
Uvaol	0.18	Literature[4]
Wogonin	0.25	Literature[10]
α-Spinasterone	0.17	Literature[11]
β-Amyrenone	-0.02	Literature[10]

Reference

1 Lee, I. K. *et al.* Triterpenoic acids of Prunella vulgaris var. lilacina and their cytotoxic activities in vitro. *Archives of pharmacal research* **31**, 1578-1583, doi:10.1007/s12272-001-2154-6 (2008).

2 Zhang, L. Z. *et al.* A novel triterpenoid saponin from Prunella vulgaris. *Acta pharmaceutica Sinica* **43**, 169-172 (2008).

3 Ju, Z. *et al.* Depsides from Prunella vulgaris. *Chinese Chemical Letters* (2000).

4 Du, D., Cheng, Z. & Chen, D. A new unusual delta11(12)-oleane triterpene and anti-complementary triterpenes from Prunella vulgaris spikes. *Natural product communications* **7**, 501-505 (2012).

5 Li, H. M. *et al.* The inhibitory effect of Prunella vulgaris L. on aldose reductase and protein glycation. *Journal of biomedicine & biotechnology* **2012**, 928159, doi:10.1155/2012/928159 (2012).

6 Wang, Z., Zhao, Y., Tu, G., Hong, S. & Chen, Y. Studies on the chemical constituents from Prunella vulgaris. *Acta pharmaceutica Sinica* 679-681 (1999).

7 Gu, X. J., Li, Y. B., Li, P., Qian, S. H. & Duan, J. A. Studies on chemical constituents of Prunella vulgaris. *Chinese Journal of Chinese Materia Medica* **32**, 923-926 (2007).

8 Lin, L. M. *et al.* Quantitative analysis of salviaflaside and rosmarinic acid in Prunella vulgaris. *Chinese Pharmaceutical Journal* **47**, 1204-1207 (2012).

9 Kojima, H., Sato, N., Hatano, A. & Ogura, H. Sterol glucosides from Prunella vulgaris. *Phytochemistry* **29**, 2351-2355, doi:10.1016/0031-9422(90)83073-A (1990).

10 Bai, Y. *et al.* Phytochemistry and pharmacological activities of the genus Prunella. *Food chemistry* **204**, 483-496, doi:10.1016/j.foodchem.2016.02.047 (2016).

11 Qian, Y., Jin, Q. & Shou-jin, L. Study on Chemical Constituents from Prunella vulgaris. *Chinese Journal of Experimental Traditional Medical Formulae* (2012).

Supplementary table 2 41 constituents with DL⩾0.18

**Components**	**DL**(by molsoft)	**Caco-2**	**HIA**	**PPB**
Oleanolic acid-28-O-beta-D-glucopyranoside	0.54	20.6762	83.273103	97.718022
cyanidol	0.92	0.656962	66.707957	100
oleanolic acid	0.37	21.8872	95.996305	100
Sitogluside	0.51	25.2333	90.027561	100
beta-sitosterol	0.88	52.3734	100	100
rutin	1.1	7.91267	2.861176	43.897909
kaempferol	0.77	9.57744	79.439289	89.608221
Hyperin	0.89	9.43911	11.777947	59.158494
Stigmasterol	0.73	52.3376	100	100
ursolic acid	0.65	21.8616	95.996396	100
Δ7-stigmasterol	0.4	52.2667	100	100
Astragalin	0.8	11.1458	25.171632	57.57566
luteolin	0.86	4.53973	79.427233	99.717233
Malvidin-3,5-diglucoside	0.5	3.82658	3.616024	24.069454
Peonin	0.35	3.83847	4.277323	29.768936
Vulgarsaponin B	0.32	20.2436	76.978849	93.704260
Vulgaxanthin-I	0.64	14.7932	12.473061	15.303988
arjunglucoside I	0.62	20.1256	30.738043	76.250677
isoquercetrin	0.89	9.43911	11.777947	59.158494
niga-chigoside F1	0.73	19.9571	30.783166	71.850493
poriferasterol monoglucoside	0.32	25.1584	90.573263	100
poriferasterol monoglucoside_qt	0.57	54.5969	100	100
sericoside	0.62	20.1256	30.738043	76.250677
stigmast-7-enol	0.3	52.3732	100	100
morin	0.87	17.1017	63.494428	91.625121
isoorientin	0.84	4.10267	14.986599	63.454304
luteolin-7-o-glucoside	0.86	52.3732	100	100

quercetin	0.93	3.4129	63.485215	93.236103
2α,3α-dihydroxyursa-12-en-28-oic acid	0.56	21.2642	94.278443	99.224537
Acacetin-7-O-β-d-glucopyranoside	0.72	7.7312	65.899591	68.7768
Butyl rosmarinate	0.67	20.5442	82.148559	90.415048
Ethyl rosmarinate	0.62	20.5183	78.87347	86.956605
Kaempferol-3-O-rutinoside	1.02	9.13279	6.289572	42.374587
Lupenone	0.37	49.5374	100	100
Quercetin-3-O-β-d-galactoside	0.89	9.43911	11.777947	59.158494
Rhein	0.79	2.84467	82.96139	88.519104
Rosmarinic acid	0.63	20.7246	62.487577	86.242087
Salviaflaside	0.39	17.8788	10.123803	66.86432
Stigmasterol-3-O-β-d-glucoside	0.32	25.1584	90.573263	100
Uvaol	0.18	24.6579	94.405824	100
Wogonin	0.25	4.28222	93.039468	90.447036

Supplementary table 3 379 Predicted targets of 32 primary selected constituents from STITCH and Swiss Target Prediction databases

**Components**	**Target**
Oleanolic acid-28-O-beta-D-glucopyranoside	BCL2
Oleanolic acid-28-O-beta-D-glucopyranoside	BCL2L1
Oleanolic acid-28-O-beta-D-glucopyranoside	BCL2L2
Oleanolic acid-28-O-beta-D-glucopyranoside	F2
cyanidol	CA12
cyanidol	CA1
cyanidol	CA2
cyanidol	CA3
cyanidol	CA4
cyanidol	CA6
cyanidol	CA5A
cyanidol	CA7
cyanidol	CA9

cyanidol	CA13
cyanidol	TDP1
cyanidol	CA5B
cyanidol	CA14
cyanidol	BACE1
cyanidol	BACE2
cyanidol	VEGFA
cyanidol	AHR
oleanolic acid	AKR1B10
oleanolic acid	PLA2G1B
oleanolic acid	POLB
oleanolic acid	PTPN2
oleanolic acid	PTPN1
oleanolic acid	AKR1B15
oleanolic acid	AKR1A1
oleanolic acid	AKR1B1
oleanolic acid	AKR1E2
oleanolic acid	PTPRF
oleanolic acid	ACP1
oleanolic acid	HSD11B1
oleanolic acid	PTPRD
oleanolic acid	PTPRS
oleanolic acid	HSD11B1L
oleanolic acid	UGT2B11
oleanolic acid	UGT2B10
oleanolic acid	NFE2L2
oleanolic acid	NQO1
oleanolic acid	PPARA
oleanolic acid	PTGIR
oleanolic acid	NAMPT
oleanolic acid	CASP3
oleanolic acid	TOP2A
oleanolic acid	TOP1
beta-sitosterol	TDP1
beta-sitosterol	CYP17A1
beta-sitosterol	NR1H2
beta-sitosterol	NR1H3
beta-sitosterol	HMGCR
beta-sitosterol	SREBF2
beta-sitosterol	SREBF1
beta-sitosterol	AR
beta-sitosterol	LDLR
beta-sitosterol	VLDLR
beta-sitosterol	LRP8

beta-sitosterol	ABCG8
beta-sitosterol	ABCG5
beta-sitosterol	APOE
beta-sitosterol	DHCR24
beta-sitosterol	CASP3
beta-sitosterol	ABCB11
beta-sitosterol	ICAM1
rutin	ADRA2A
rutin	ADRA2C
rutin	MBNL1
rutin	DYRK1A
rutin	ADRA2B
rutin	MBNL2
rutin	MBNL3
rutin	AKR1B1
rutin	AKR1B15
rutin	AKR1B10
rutin	TDP1
rutin	NOX4
rutin	AKR1A1
rutin	AKR1E2
rutin	NQO2
rutin	AKR1C3
rutin	P4HB
rutin	EGFR
rutin	GSR
rutin	PRNP
rutin	CTGF
rutin	SREBF1
rutin	HSPA4
rutin	HSPA4
rutin	ALDH2
arjunglucoside I	F2
kaempferol	CYP1A2
kaempferol	ABCB1
kaempferol	ALOX5
kaempferol	HSD17B1
kaempferol	AKR1B1
kaempferol	ALOX15
kaempferol	ALOX12
kaempferol	ABCC1
kaempferol	AHR
kaempferol	HSD17B2
kaempferol	XDH

kaempferol	DYRK1A
kaempferol	CYP1B1
kaempferol	NOX4
kaempferol	TDP1
kaempferol	CDK1
kaempferol	NR1I2
kaempferol	UGT3A1
kaempferol	RPS6KA3
kaempferol	UGT1A3
kaempferol	UGT1A8
kaempferol	UGT1A7
kaempferol	UGT1A9
Stigmasterol	TDP1
Stigmasterol	NR1H2
Stigmasterol	NR1H3
Stigmasterol	CYP17A1
Stigmasterol	HMGCR
Stigmasterol	CYP51A1
Stigmasterol	SREBF2
Stigmasterol	SREBF1
Stigmasterol	AR
Stigmasterol	LDLR
Stigmasterol	VLDLR
Stigmasterol	LRP8
Stigmasterol	ABCA1
Stigmasterol	ABCG8
Stigmasterol	ABCG5
Stigmasterol	IL10
Stigmasterol	TNF
Stigmasterol	IL8
Stigmasterol	SLCO1B1
ursolic acid	AKR1B10
ursolic acid	POLB
ursolic acid	PTPRF
ursolic acid	PTPN2
ursolic acid	PTPN1
ursolic acid	ACP1
ursolic acid	HSD11B1
ursolic acid	AKR1B15
ursolic acid	AKR1A1
ursolic acid	AKR1B1
ursolic acid	Q96JD6
ursolic acid	PTPRD
ursolic acid	PTPRS

ursolic acid	HSD11B1L
ursolic acid	PLA2G1B
ursolic acid	TOP2A
ursolic acid	CYP2C19
ursolic acid	ERN1
ursolic acid	PARP1
ursolic acid	MTOR
ursolic acid	TOP1
ursolic acid	BAK1
ursolic acid	CASP8
ursolic acid	ACACA
ursolic acid	CTNNB1
Astragalin	AKR1B1
Astragalin	AKR1B15
Astragalin	AKR1B10
Astragalin	DYRK1A
Astragalin	AKR1A1
Astragalin	AKR1E2
Astragalin	TDP1
Astragalin	NQO2
Astragalin	NQO1
Astragalin	ADRA2A
Astragalin	ADRA2C
Astragalin	NOX4
Astragalin	ADRA2B
Astragalin	MBNL1
Astragalin	MBNL2
Astragalin	PTGS2
luteolin	MMP1
luteolin	CYP1A2
luteolin	MMP2
luteolin	MMP3
luteolin	MMP9
luteolin	AKR1B1
luteolin	MAOA
luteolin	MAOB
luteolin	CD38
luteolin	ADORA1
luteolin	MMP12
luteolin	MMP13
luteolin	XDH
luteolin	GLO1
luteolin	KARS
luteolin	MAPK8

luteolin	CASP3
luteolin	JUN
luteolin	FOS
luteolin	CDK2
luteolin	EGFR
luteolin	SMAD2
luteolin	CCNA2
luteolin	AKT1
nigaichigoside F1	F2
sericoside	F2
stigmast-7-enol	TDP1
stigmast-7-enol	CYP17A1
stigmast-7-enol	NR1H2
stigmast-7-enol	NR1H3
stigmast-7-enol	SLC6A4
stigmast-7-enol	SLC6A2
stigmast-7-enol	SLC6A3
stigmast-7-enol	SLC6A9
stigmast-7-enol	SLC6A7
stigmast-7-enol	SLC6A14
stigmast-7-enol	SLC6A5
stigmast-7-enol	BCHE
stigmast-7-enol	ACHE
stigmast-7-enol	HMGCR
stigmast-7-enol	SREBF2
morin	ESR1
morin	ESR2
morin	SLC22A12
morin	GPR35
morin	TDP1
morin	SLC22A10
morin	SLC22A25
morin	SLC22A9
morin	SLC22A24
morin	SLC22A11
morin	CYP1A2
morin	ALOX5
morin	AKR1B1
morin	ALOX15
morin	ALOX12
morin	CASP3
morin	ABCB1
morin	HMOX1
morin	CASP7

morin	NR0B2
morin	CASP9
morin	ABCC1
morin	ABCB11
morin	UGT1A8
morin	UGT1A7
luteolin-7-glucoside	NOS3
luteolin-7-glucoside	TDP1
luteolin-7-glucoside	HMOX1
luteolin-7-glucoside	NOS2
luteolin-7-glucoside	KDM4A
luteolin-7-glucoside	KDM4B
luteolin-7-glucoside	KDM4C
luteolin-7-glucoside	AKR1B10
luteolin-7-glucoside	AKR1B1
luteolin-7-glucoside	AKR1B15
luteolin-7-glucoside	XDH
luteolin-7-glucoside	AOX1
luteolin-7-glucoside	ADORA1
luteolin-7-glucoside	MBNL1
luteolin-7-glucoside	MBNL2
luteolin-7-glucoside	MBNL3
quercetin	CA12
quercetin	EGFR
quercetin	CA1
quercetin	CA2
quercetin	PLA2G1B
quercetin	ERBB2
quercetin	MPO
quercetin	CYP1A2
quercetin	CDK1
quercetin	CA3
quercetin	PRSS1
quercetin	MMP2
quercetin	MMP3
quercetin	ALOX5
quercetin	MAPT
quercetin	MCL1
quercetin	CYP1B1
quercetin	HCK
quercetin	PIM1
quercetin	SLC2A2
quercetin	CYP2C8
quercetin	CYP1A1

quercetin	ATP5B
quercetin	HIBCH
quercetin	STK17B
Rosmarinicacid	AKR1B10
Rosmarinicacid	FYN
Rosmarinicacid	SRC
Rosmarinicacid	AKR1B1
Rosmarinicacid	TDP1
Rosmarinicacid	AKR1B15
Rosmarinicacid	AKR1A1
Rosmarinicacid	AKR1E2
Rosmarinicacid	YES1
Rosmarinicacid	FGR
Rosmarinicacid	FRK
Rosmarinicacid	MMP1
Rosmarinicacid	MMP2
Rosmarinicacid	MMP3
Rosmarinicacid	MMP9
Uvaol	CHRM2
Uvaol	CHRM4
Uvaol	NR1H2
Uvaol	NR1H3
Uvaol	ACHE
Uvaol	BCHE
Uvaol	SLC6A2
Uvaol	SLC6A4
Uvaol	SLC6A3
Uvaol	CHRM1
Uvaol	CHRM3
Uvaol	CHRM5
Uvaol	AR
Uvaol	SLC6A9
Uvaol	SLC6A5
Wogonin	PTGS1
Wogonin	NOS2
Wogonin	PTGS2
Wogonin	NOS3
Wogonin	NOS1
Wogonin	AKR1B1
Wogonin	AKR1B15
Wogonin	AKR1B10
Wogonin	IKBKB
Wogonin	CHUK
Wogonin	DYRK1A

Wogonin	MAPT
Wogonin	ABCG2
Wogonin	MMP9
Wogonin	CDK9
Wogonin	MCL1
Wogonin	GATA1
Wogonin	CCL2
Wogonin	KCNK10
Wogonin	MYC
Wogonin	PLSCR1
Wogonin	HMGB1
Acacetin-7-O-β-d-glucopyranoside	ADORA1
Acacetin-7-O-β-d-glucopyranoside	TDP1
Ethyl rosmarinate	MMP1
Ethyl rosmarinate	MMP2
Ethyl rosmarinate	MMP3
Ethyl rosmarinate	MMP10
Ethyl rosmarinate	MMP9
Ethyl rosmarinate	MMP12
Ethyl rosmarinate	MMP13
Ethyl rosmarinate	MMP27
Ethyl rosmarinate	TDP1
Ethyl rosmarinate	AKR1B10
Ethyl rosmarinate	AKR1B1
Ethyl rosmarinate	AKR1B15
Ethyl rosmarinate	AKR1A1
Ethyl rosmarinate	AKR1E2
Ethyl rosmarinate	FYN
Butyl rosmarinate	MMP1
Butyl rosmarinate	MMP2
Butyl rosmarinate	MMP3
Butyl rosmarinate	MMP10
Butyl rosmarinate	MMP9
Butyl rosmarinate	MMP12
Butyl rosmarinate	MMP13
Butyl rosmarinate	MMP27
Butyl rosmarinate	FYN
Butyl rosmarinate	YES1
Butyl rosmarinate	FGR
Butyl rosmarinate	SRC
Butyl rosmarinate	FRK
Butyl rosmarinate	AKR1B10
Butyl rosmarinate	AKR1B1
Rhein	MBNL1

Rhein	TDP1
Rhein	MBNL2
Rhein	MBNL3
Rhein	MAPT
Rhein	UCP1
Rhein	PPARG
Rhein	FTO
Rhein	RXRA
Rhein	PPARA
Rhein	DNM1L
Rhein	VEGFA
Rhein	RELA
Rhein	NFKB1
Sitogluside	——
Δ7-stigmasterol	——
Vulgarsaponin B	——
Poriferasterol monoglucoside	——
Poriferasterol monoglucoside_qt	——
2α,3α-dihydroxyursa-12-en-28-oic acid	——
Lupenone	——
Stigmasterol-3-O-β-d-glucoside	——

Supplementary table 4 204 genes associated with breast cancer acquired from Malacards and GeneCards

Target gene	Target gene	Target gene
TP53	SRC	MIR502
BRCA2	AXIN2	MIR520C
BRCA1	PMS2	ABRAXAS1
CDH1	EP300	NQO2
ERBB2	H19	XRCC2
PTEN	MUC1	KLLN
MLH1	FHIT	CASP3
MSH2	CXCR4	TWIST1
APC	AKT2	PDGFRB
ESR1	RASSF1	FLT1
PIK3CA	PPM1D	PTGS2
CCND1	GNAS	BMPR1A
MSH6	AURKA	RAD51L3-RFFL
ATM	ATR	WWOX
AKT1	TGFBR1	FGFR4
KRAS	CYP17A1	CDKN3
RB1	PRLR	IGF1
CHEK2	TOP2A	GNRH1
VEGFA	EGF	IGF1R
CTNNB1	XRCC3	KRT8
MYC	NME1	MMP9
PALB2	MMP1	MIR145
MET	TP63	INS
AR	KRT5	MUTYH
STK11	KRT14	MAPK1
BRAF	PHB	GATA3
TGFB1	RAD54L	MIR143
CDKN1A	HMMR	MMP2
HRAS	SLC22A18	GLI1
STAT3	RB1CC1	CXCL12
SMAD4	MIR155	CYP1B1
CDKN1B	MIR221	HIF1A
TERT	MIR146A	TCF7L2
CDK4	MIR222	FOXO1
TNF	MIR10B	ABCG2
BAX	MIR205	ABCB1
CDKN2A	IDH1	SPP1
BRIP1	MIR206	MIR203A
NF1	MIR661	GSTP1
MDM2	MIR196A2	ERBB4

TGFBR2	MIR31	CTSD
TP53	MIR204	SNAI2
EGFR	MIR125A	CD44
BARD1	MIR127	NRAS
RAD51D	HERC2	BIRC5
NBN	MIR210	JUN
RAD51C	MIR200C	ODC1
CYP19A1	MIR34A	MIR195
FGFR2	MIR200B	NOS2
MRE11	MIR27A	TFF1
RAD51	MIR200A	HSPB1
POLE	MIR429	RET
IL6	MIR451A	SMAD7
CASP8	MIR199B	PPARG
ESR2	MIR126	BCAR3
PGR	MIR335	MKI67
MTOR	MIR373	FGFR3
RAD50	MIR146B	IRS1
PLAU	MIR128-1	ERBB3
NOTCH1	MIR20A	FGF8
FGFR1	MIR510	MIR148A
IGF2	MIR9-3	AHR
KIT	MIR141	PARP1
MIR21	MIR193B	RARA
BCL2	MIR96	BCAR1
EPCAM	MIR182	C11orf65
TYMS	MIR30E	MIR191
ALK	MIR499A	WNT5A

Supplementary table 5 top 20 GO enrichment

Description	p.adjust	geneID	Count
estrogen receptor binding	1.77313E-06	ESR1/CTNNB1/SRC/PPARG/PARP1	5
nuclear receptor activity	1.87986E-06	ESR1/AR/ESR2/PPARG/AHR	5
transcription factor activity, direct ligand regulated sequence-specific DNA binding	1.87986E-06	ESR1/AR/ESR2/PPARG/AHR	5
protein phosphatase binding	4.82447E-06	ERBB2/AKT1/CTNNB1/EGFR/BCL2/PPARG	6
phosphatidylinositol-4,5-bisphosphate 3-kinase activity	4.82447E-06	ERBB2/ESR1/EGFR/SRC/EGF	5
RNA polymerase II transcription factor binding	4.82447E-06	ESR1/CTNNB1/AR/JUN/PPARG/AHR	6
phosphatidylinositol bisphosphate kinase activity	4.82447E-06	ERBB2/ESR1/EGFR/SRC/EGF	5
phosphatidylinositol 3-kinase activity	7.23567E-06	ERBB2/ESR1/EGFR/SRC/EGF	5
steroid hormone receptor binding	1.23734E-05	ESR1/CTNNB1/SRC/PPARG/PARP1	5
phosphatase binding	1.36507E-05	ERBB2/AKT1/CTNNB1/EGFR/BCL2/PPARG	6
nitric-oxide synthase regulator activity	1.71818E-05	ESR1/AKT1/EGFR	3
steroid hormone receptor activity	4.55279E-05	ESR1/AR/ESR2/PPARG	4
heme binding	5.51499E-05	SRC/CYP17A1/PTGS2/CYP1B1/NOS2	5
tetrapyrrole binding	7.34774E-05	SRC/CYP17A1/PTGS2/CYP1B1/NOS2	5
RNA polymerase II basal transcription factor binding	8.61259E-05	ESR1/AR/AHR	3
nuclear hormone receptor binding	9.28269E-05	ESR1/CTNNB1/SRC/PPARG/PARP1	5
activating transcription factor binding	0.000126907	CTNNB1/MYC/JUN/PPARG	4
hormone receptor binding	0.000213669	ESR1/CTNNB1/SRC/PPARG/PARP1	5
protein C-terminus binding	0.000213669	ERBB2/CTNNB1/SRC/TOP2A/PPARG	5
cofactor binding	0.000226723	SRC/CYP17A1/NQO2/PTGS2 /CYP1B1/NOS2/PARP1	7

Supplementary table 6 top 20 KEGG pathway

**Description**	**p.adjust**	**geneID**	**Count**
Bladder cancer	2.60548E-12	ERBB2/VEGFA/MYC/EGFR/SRC/EGF/MMP1/MMP9/MMP2	9
Endocrine resistance	2.60548E-12	ERBB2/ESR1/AKT1/EGFR/ESR2/MTOR/BCL2/SRC/MMP9/MMP2/JUN	11
Proteoglycans in cancer	6.77004E-12	ERBB2/ESR1/AKT1/VEGFA/CTNNB1/MYC/TNF/EGFR/MTOR/SRC/CASP3/MMP9/MMP2	13
Colorectal cancer	7.85973E-10	AKT1/CTNNB1/MYC/EGFR/MTOR/BCL2/EGF/CASP3/JUN	9
Prostate cancer	1.90246E-09	ERBB2/AKT1/CTNNB1/AR/EGFR/MTOR/BCL2/EGF/MMP9	9
Breast cancer	2.53884E-09	ERBB2/ESR1/AKT1/CTNNB1/MYC/EGFR/ESR2/MTOR/EGF/JUN	10
Human cytomegalovirus infection	7.28818E-09	AKT1/VEGFA/CTNNB1/MYC/TNF/EGFR/CASP8/MTOR/SRC/CASP3/PTGS2	11
EGFR tyrosine kinase inhibitor resistance	8.00229E-09	ERBB2/AKT1/VEGFA/EGFR/MTOR/BCL2/SRC/EGF	8
ErbB signaling pathway	1.29246E-08	ERBB2/AKT1/MYC/EGFR/MTOR/SRC/EGF/JUN	8
Relaxin signaling pathway	1.35201E-08	AKT1/VEGFA/EGFR/SRC/MMP1/MMP9/MMP2/JUN/NOS2	9
Kaposi sarcoma-associated herpesvirus infection	1.4202E-08	AKT1/VEGFA/CTNNB1/MYC/CASP8/MTOR/SRC/CASP3/PTGS2/JUN	10
Estrogen signaling pathway	1.89344E-08	ESR1/AKT1/EGFR/ESR2/BCL2/SRC/MMP9/MMP2/JUN	9
Fluid shear stress and atherosclerosis	1.89344E-08	AKT1/VEGFA/CTNNB1/TNF/BCL2/SRC/MMP9/MMP2/JUN	9
HIF-1 signaling pathway	3.04704E-08	ERBB2/AKT1/VEGFA/EGFR/MTOR/BCL2/EGF/NOS2	8
Gastric cancer	3.04704E-08	ERBB2/AKT1/CTNNB1/MYC/EGFR/MTOR/BCL2/EGF/ABCB1	9
Hepatitis B	6.1543E-08	AKT1/MYC/TNF/CASP8/BCL2/SRC/CASP3/MMP9/JUN	9
MicroRNAs in cancer	6.1543E-08	ERBB2/VEGFA/MYC/EGFR/MTOR/BCL2/CASP3/PTGS2/MMP9/CYP1B1/ABCB1	11
Focal adhesion	3.23356E-07	ERBB2/AKT1/VEGFA/CTNNB1/EGFR/BCL2/SRC/EGF/JUN	9
IL-17 signaling pathway	3.98581E-07	TNF/CASP8/MMP1/CASP3/PTGS2/MMP9/JUN	7
Endometrial cancer	5.74642E-07	ERBB2/AKT1/CTNNB1/MYC/EGFR/EGF	6

Supplementary table 7 PPI result from STRING database

node1	node2	combined_score
CASP8	CASP3	0.999
AKT1	MTOR	0.999
EGFR	EGF	0.999
AKT1	CTNNB1	0.997
BCL2	CASP8	0.996
ERBB2	EGF	0.996
VEGFA	EGF	0.993
MYC	AKT1	0.992
MYC	JUN	0.992
MYC	CTNNB1	0.991
AKT1	ESR1	0.991
AR	SRC	0.99
ESR1	SRC	0.99
PARP1	CASP3	0.99
SRC	EGF	0.99
CTNNB1	EGFR	0.99
VEGFA	MMP9	0.989
CTNNB1	ERBB2	0.989
SRC	CTNNB1	0.988
VEGFA	SRC	0.987
TNF	JUN	0.986
TNF	CASP8	0.986
MTOR	EGFR	0.986
AR	CTNNB1	0.984
TNF	NOS2	0.984
VEGFA	JUN	0.983
MYC	ESR1	0.983
CTNNB1	CASP3	0.982
TNF	PPARG	0.982
JUN	CTNNB1	0.981
SRC	MMP9	0.979
ESR1	JUN	0.979
VEGFA	EGFR	0.979
VEGFA	CTNNB1	0.979
VEGFA	MMP2	0.979
AKT1	SRC	0.978
CTNNB1	EGF	0.978
MYC	TNF	0.976
SRC	NOS2	0.974

SRC	MMP2	0.974
EGFR	ERBB2	0.971
SRC	JUN	0.97
AKT1	CASP3	0.969
VEGFA	AKT1	0.966
AR	CASP8	0.965
SRC	ESR2	0.965
MYC	BCL2	0.965
MTOR	EGF	0.965
SRC	EGFR	0.963
MMP9	MMP1	0.961
SRC	ERBB2	0.961
JUN	NOS2	0.956
JUN	PPARG	0.956
VEGFA	ERBB2	0.956
ESR1	ERBB2	0.947
TNF	MMP9	0.944
ESR1	EGFR	0.943
CYP1B1	AHR	0.943
AKT1	BCL2	0.941
ESR1	ESR2	0.941
AKT1	EGF	0.933
AKT1	AR	0.932
TNF	PTGS2	0.926
ESR1	BCL2	0.925
MMP1	MMP2	0.922
VEGFA	TNF	0.919
VEGFA	PTGS2	0.918
MYC	NOS2	0.918
AKT1	JUN	0.913
AKT1	NOS2	0.909
MMP9	MMP2	0.908
PTGS2	NOS2	0.906
MTOR	PPARG	0.901
MYC	ERBB2	0.895
MMP9	JUN	0.893
ESR1	AHR	0.888
AKT1	MMP9	0.887
JUN	ESR2	0.882
JUN	EGFR	0.88
MYC	EGF	0.877

AKT1	TNF	0.876
JUN	CASP3	0.874
VEGFA	MTOR	0.873
TNF	CASP3	0.873
MYC	EGFR	0.872
PTGS2	EGFR	0.872
MMP9	EGF	0.871
PTGS2	EGF	0.866
MYC	VEGFA	0.854
AKT1	PTGS2	0.854
MYC	AR	0.853
CTNNB1	PPARG	0.842
ESR1	CTNNB1	0.842
AR	ERBB2	0.84
TNF	EGF	0.839
MYC	SRC	0.838
JUN	EGF	0.837
PTGS2	CTNNB1	0.837
ESR1	EGF	0.836
AKT1	MMP2	0.836
VEGFA	MMP1	0.832
MTOR	ERBB2	0.83
AKT1	ESR2	0.828
MMP9	EGFR	0.828
PTGS2	ERBB2	0.822
PARP1	CASP8	0.82
PTGS2	PPARG	0.819
VEGFA	ESR1	0.816
MTOR	CASP3	0.811
AR	EGF	0.81
AR	CYP17A1	0.807
AR	EGFR	0.803
TNF	MMP2	0.798
TNF	MMP1	0.798
EGF	MMP2	0.798
AKT1	PARP1	0.791
PPARG	EGFR	0.787
MMP9	ERBB2	0.781
SRC	CASP8	0.779
MYC	CASP3	0.775
JUN	MTOR	0.774

ESR1	PTGS2	0.773
MMP1	EGF	0.771
VEGFA	PPARG	0.769
JUN	MMP1	0.767
AKT1	ABCG2	0.763
BCL2	CASP3	0.763
ESR1	PARP1	0.761
ESR1	MTOR	0.759
ERBB2	MMP2	0.758
MYC	PARP1	0.755
ABCB1	EGFR	0.754
CASP3	EGFR	0.751
ESR2	EGFR	0.749
AR	CASP3	0.746
AKT1	CASP8	0.746
VEGFA	AHR	0.744
VEGFA	AR	0.742
MMP9	PTGS2	0.741
VEGFA	CASP3	0.737
AKT1	ERBB2	0.737
AR	JUN	0.736
CASP8	EGFR	0.736
AR	AHR	0.736
MYC	MMP2	0.734
EGF	ABCG2	0.724
MMP9	CASP3	0.723
MMP1	ERBB2	0.721
AR	MMP9	0.717
TNF	EGFR	0.716
MTOR	CTNNB1	0.712
CYP1B1	ESR2	0.711
JUN	PTGS2	0.711
MMP9	PPARG	0.709
JUN	ERBB2	0.708
MYC	ABCG2	0.706
PTGS2	MMP2	0.704
CASP3	EGF	0.698
EGFR	MMP2	0.697
AHR	ABCG2	0.697
PTGS2	MMP1	0.696
AKT1	PPARG	0.695

TNF	AHR	0.695
CASP3	ERBB2	0.694
JUN	CASP8	0.692
PTGS2	AHR	0.69
SRC	MTOR	0.688
ESR1	ABCG2	0.685
MYC	MTOR	0.685
PTGS2	CASP3	0.685
SRC	CASP3	0.683
AKT1	EGFR	0.678
CYP1B1	PTGS2	0.678
PPARG	AHR	0.677
MYC	ABCB1	0.675
CYP1B1	ESR1	0.673
ESR2	ERBB2	0.671
VEGFA	ESR2	0.671
TOP2A	ERBB2	0.67
CASP3	MMP2	0.669
MTOR	MMP2	0.667
ESR1	CASP3	0.665
MYC	MMP9	0.664
EGFR	ABCG2	0.661
PPARG	MMP2	0.658
JUN	PARP1	0.655
PPARG	EGF	0.65
ABCB1	SRC	0.646
EGFR	AHR	0.643
JUN	MMP2	0.64
MYC	CASP8	0.639
TNF	AR	0.639
MYC	AHR	0.638
TNF	SRC	0.637
ESR1	MMP1	0.633
MMP9	CTNNB1	0.633
SRC	AHR	0.628
ESR1	CYP17A1	0.627
CASP8	CTNNB1	0.619
ABCB1	CTNNB1	0.612
CTNNB1	MMP2	0.604
CYP17A1	ESR2	0.597
JUN	ABCG2	0.597

PPARG	ERBB2	0.596
ABCB1	AKT1	0.594
TNF	ERBB2	0.588
PPARG	ABCG2	0.588
ESR1	AR	0.587
MTOR	CASP8	0.584
ABCB1	TNF	0.583
ESR1	MMP9	0.581
SRC	PTGS2	0.581
CASP3	PPARG	0.578
TNF	MTOR	0.575
PARP1	EGFR	0.573
CTNNB1	AHR	0.573
MYC	PTGS2	0.57
MMP1	PPARG	0.567
MYC	TOP2A	0.566
MMP9	AHR	0.56
VEGFA	CASP8	0.559
ERBB2	ABCG2	0.556
ESR1	TNF	0.553
TOP2A	CTNNB1	0.551
AR	MTOR	0.55
AKT1	MMP1	0.547
ABCB1	ERBB2	0.541
MMP1	EGFR	0.541
ABCB1	CASP3	0.541
VEGFA	NOS2	0.538
AKT1	NQO2	0.534
NOS2	CASP3	0.533
ESR1	MMP2	0.532
MMP9	NOS2	0.53
CASP8	ERBB2	0.53
TOP2A	EGFR	0.53
PARP1	CTNNB1	0.527
VEGFA	ABCG2	0.527
ESR2	ABCG2	0.525
PARP1	NOS2	0.523
SRC	CYP17A1	0.521
PARP1	ERBB2	0.519
CASP8	EGF	0.519
ESR2	AHR	0.519

PTGS2	CASP8	0.518
MMP1	AHR	0.518
MMP9	CASP8	0.516
ABCB1	PTGS2	0.514
BCL2	PARP1	0.514
ESR1	CASP8	0.509
AR	MMP1	0.509
VEGFA	ABCB1	0.509
NOS2	AHR	0.505
ABCB1	ABCG2	0.498
MYC	PPARG	0.496
PTGS2	MTOR	0.493
CYP1B1	MMP2	0.491
TOP2A	JUN	0.49
TOP2A	ESR1	0.488
TNF	CTNNB1	0.483
MMP9	MTOR	0.48
ESR2	EGF	0.473
SRC	PARP1	0.472
PARP1	MTOR	0.469
CYP1B1	ABCB1	0.461
ABCB1	ESR1	0.46
NOS2	PPARG	0.459
CASP8	MMP2	0.456
MMP1	CASP3	0.453
ESR2	CASP3	0.452
CASP3	ABCG2	0.451
TNF	PARP1	0.447
AKT1	AHR	0.447
AR	PARP1	0.445
PTGS2	ESR2	0.444
CTNNB1	ABCG2	0.443
ABCB1	EGF	0.443
AR	PTGS2	0.442
ABCB1	TOP2A	0.439
SRC	MMP1	0.431
JUN	AHR	0.43
MYC	ESR2	0.429
CYP1B1	ABCG2	0.423
TOP2A	PARP1	0.42
AR	ABCG2	0.42

CTNNB1	ESR2	0.416
ABCB1	JUN	0.415
AR	MMP2	0.412
SRC	PPARG	0.411
MYC	MMP1	0.408
SRC	ABCG2	0.403
VEGFA	PARP1	0.4
